# Ungewöhnliche Präsentation einer Psoriasis vulgaris bei einem 11-jährigen Patienten

**DOI:** 10.1007/s00105-021-04933-y

**Published:** 2022-01-05

**Authors:** Viktoria Gruber, Wolfgang Weger, Lorenzo Cerroni, Barbara Binder

**Affiliations:** Universitätsklinik für Dermatologie und Venerologie, Medizinische Universität Graz, Auenbruggerplatz 8, 8036 Graz, Österreich

**Keywords:** Pädiatrie, Psoriasis vulgaris, Klinische Varianten, Differenzialdiagnosen, Therapieoptionen, Pediatrics, Psoriasis vulgaris, Clinical variants, Differential diagnosis, Treatment options

## Abstract

Die Psoriasis vulgaris tritt mit einer Prävalenz von bis zu 2 % im Kindes- und Jugendalter auf. Größtenteils wird die Diagnose klinisch gestellt. Wir berichten über einen pädiatrischen Patienten, welcher uns aufgrund der für eine Psoriasis vulgaris eher untypischen Lokalisation vor eine diagnostische Herausforderung stellte. Diskutiert werden die wichtigsten Differenzialdiagnosen der verschiedenen Psoriasisformen sowie die aktuellen Therapieempfehlungen im Kindes- und Jugendalter.

## Abstract (en)

Psoriasis vulgaris occurs with a prevalence of up to 2% in childhood and adolescence. In most cases, diagnosis can be made clinically. We report about a pediatric patient with psoriasis vulgaris who posed a diagnostic challenge due to uncharacteristic manifestation. The most important differential diagnoses of various forms of psoriasis vulgaris as well as actual treatment recommendations in childhood and adolescence are discussed.

## Anamnese

Ein 11-jähriger Patient stellte sich in Begleitung seiner Mutter in der Ambulanz für pädiatrische Dermatologie aufgrund juckender therapieresistenter Hautveränderungen am Gesäß seit 4 Jahren vor. Die bisherigen lokalen Behandlungen umfassten blande Externa, Antimykotika, Glukokortikoide sowie Antibiotika. Der Patient nahm keine Dauermedikation ein. In der Krankengeschichte fanden sich neben einer Phimose im Kleinkindalter und einer Schädelprellung nach einem Fahrradsturz keine Besonderheiten. In der Familienanamnese war zu erheben, dass sowohl ein Onkel als auch eine Cousine des Jungen an einer Psoriasis vulgaris litten.

### Klinischer Befund

**Figure 1:**
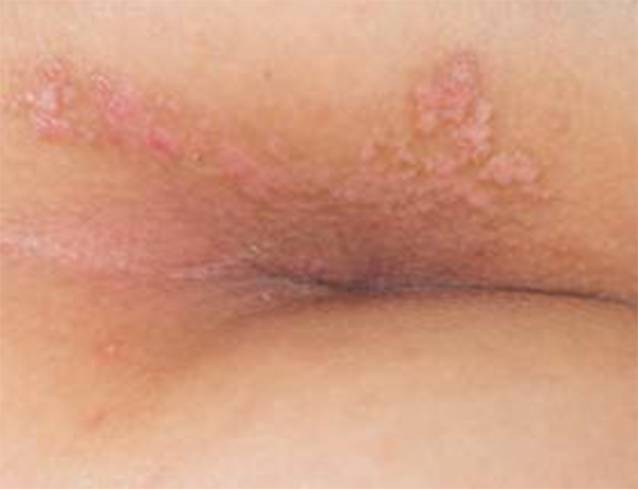

In der dermatologischen Untersuchung zeigten sich in der Gesäßfalte und der rechtsseitigen Glutealregion mehrere hautfarbene bis blassrote, teils konfluierende kleinpapulöse Plaques mit teilweise nur diskreter weißlicher Schuppung und stellenweise oberflächlichen Exkoriationen (Abb. 1).

### Diagnose

**Figure 2:**
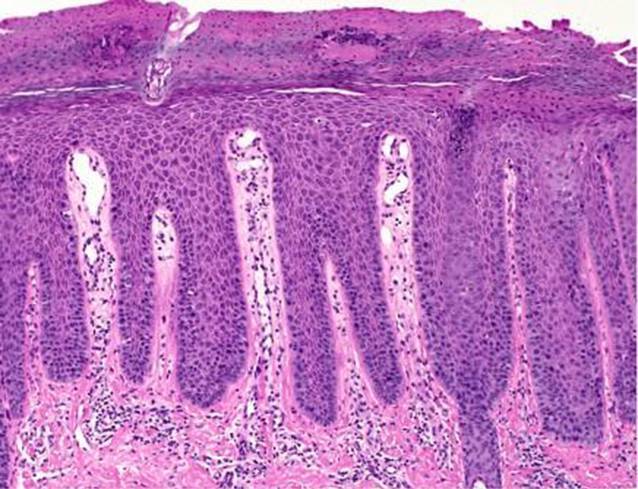

Zur weiterführenden Diagnostik wurde eine Probebiopsie (4-mm-Stanze) in Lokalanästhesie entnommen (Abb. 2).

## Therapie und Verlauf

Unser Patient hat eine kurzfristige Lokaltherapie mit einem Glukokortikoid der Klasse III erhalten, welches im Anschluss durch ein topisches Vitamin-D-Derivat ersetzt wurde. Vier Wochen nach Einleitung der Therapie war der störende Juckreiz bereits abgeklungen, und die Hautveränderungen sind flacher geworden. Im Rahmen der Langzeitbetreuung werden an unserer Klinik Kontrollen in 3‑monatigen Abständen durchgeführt.

## Diskussion

Die Psoriasis vulgaris tritt sowohl im Kindes- als auch Erwachsenenalter in etwa 70 % der Fälle als Plaquepsoriasis auf [11]. Die Schuppung und Infiltration der Herde können jedoch bei Kindern milder als bei Erwachsenen ausgeprägt sein [3].

Etwa 30 % der Kinder mit Psoriasis vulgaris präsentieren sich initial mit einer Psoriasis guttata. Diese akute Verlaufsform tritt häufig in Assoziation mit einer Infektion durch β‑hämolysierende Streptokokken oder Viren auf. In diesem Zusammenhang zählen die Angina tonsillaris als auch die streptogene perianale Dermatitis zu den häufigsten Auslösern [11].

Seltenere Varianten beinhalten unter anderem die Psoriasis inversa, Windelpsoriasis, Psoriasis pustulosa, Psoriasis palmoplantaris und die Erythrodermie [7].

Eine Besonderheit bei pädiatrischen Patienten mit Psoriasis vulgaris stellt die bevorzugte Beteiligung bestimmter Hautareale wie etwa Gesicht, Capillitium, Intertrigines und Windelbereich dar. Im Gegensatz zur irritativen Windeldermatitis sind bei der Windelpsoriasis auch die Inguinalfalten mit betroffen. Eine Beteiligung von Nägeln oder Gelenken wird bei jeweils bis zu 40 % der Betroffenen beobachtet [12].

In der Regel wird die Diagnose einer Psoriasis vulgaris anhand des typischen klinischen Erscheinungsbildes gestellt. Dennoch können seltenere Psoriasisvarianten und das Auftreten psoriatischer Hautveränderungen an atypischen anatomischen Lokalisationen eine diagnostische Herausforderung darstellen. Bei nicht eindeutigem klinischem Befund ist eine Probebiopsie zur histologischen Diagnosesicherung notwendig.

Die Differenzialdiagnosen umfassen unter anderem das seborrhoische und atopische Ekzem, die Pityriasis rosea, Epidermomykosen oder den Morbus Leiner (Tab. 1).

**Table 1:** 

Psoriasisform	Differenzialdiagnosen mit Unterscheidungsmerkmalen
*Psoriasis vulgaris (nummularis)*	*Seborrhoisches Ekzem*
	Erythematöse Plaques mit gelblich-fettiger Schuppung in talgdrüsenreichen Arealen (Kopfhaut, äußeres Ohr, zentrales Gesicht, oberer Stamm, Intertrigines)
	Fehlen von Nagelveränderungen
	*Atopisches Ekzem*
	Unscharf begrenzte erythematöse Plaques und exkoriierte Papeln
	Juckreiz meist stärker als bei der Psoriasis ausgeprägt
	Aussparung der Windelregion, Assoziation mit Allergien und Asthma bronchiale
	*Nummuläres Ekzem*
	Relativ scharf begrenzte, runde erythematöse Plaques mit Schuppung oder feinen Fissuren zwischen 1 und 10 cm Durchmesser an Stamm und Extremitäten
	Aussparung von Gesicht und Kopfhaut
	*Erythrokeratodermia symmetrica progressiva*
	Scharf begrenzte erythematöse hyperkeratotische Plaques an Streckseiten der Extremitäten
*Psoriasis guttata (exanthematica)*	*Pityriasis rosea*
	Akutes Auftreten multipler erythematöser Papeln und Plaques mit Collerette-Schuppung an Stamm und proximalen Extremitäten, häufig Beginn mit „Primärmedaillon“
	Meist Aussparung des Gesichts und der Intertrigines
	*Tinea corporis*
	Scharf begrenzte rundovale Erytheme oder Plaques mit randbetonter Schuppung oder pustulösem Randsaum und zentralem Auslöschphänomen
	Erregernachweis kulturell
	*Pityriasis lichenoides chronica*
	Chronisch rezidivierender Verlauf mit Auftreten disseminierter erythematöser Papeln mit zentraler Deckelschuppe an Stamm und Extremitäten
	Aussparung des Kopfes
*Psoriasis inversa*	*Intertriginöse Kandidose*
	Erytheme mit abstreifbaren weißen Belägen, im Randbereich papulopustulöse Satelliten
	Erregernachweis mikroskopisch, kulturell
	*Irritative Windeldermatitis*
	Scharf begrenzte Rötungen und erythematöse Papeln mit Erosionen oder Mazeration an Gesäß, Genitale, Unterbauch und Oberschenkeln
	Aussparung der Hautfalten
*Erythrodermatische Psoriasis*	*Kutane T‑Zell-Lymphome*
	Erythrodermie und Schuppung mit Lymphadenopathie
	Diagnosesicherung histologisch, immunhistologisch
	*Morbus Leiner*
	Maximalvariante des seborrhoischen Säuglingsekzems mit Erythrodermie und fettig-lamellöser Schuppung
	Diarrhö, Infektneigung und Gedeihstörungen
	*Kongenitale ichthyosiforme Erythrodermie*
	Erythrodermie mit heller feiner Schuppung („Kollodiumbaby“)
	Gestörte Temperaturregulation mit Neigung zu Hyperthermie
*Psoriasis palmoplantaris*	*Tinea manuum et pedum*
	Palmoplantare Erytheme, Schuppen mit teilweise Bläschen oder Rhagaden
	Erregernachweis kulturell
*Psoriasis capitis*	*Seborrhoisches Säuglingsekzem*
	Gelblich-fettige Schuppung an der Kopfhaut (frontoparietal) mit mildem Juckreiz, wobei die Stirn‑/Nackenhaargrenze nicht überschritten wird
	Häufig Beginn im Gesicht mit erythematösen schuppenden Plaques
	*Tinea capitis (Mikrosporie)*
	Meist juckende Alopezieherde mit feinlamellärer Schuppung und Erythem
	Erregernachweis mikroskopisch, kulturell
	*Pityriasis (Tinea) amiantacea*
	Festhaftende asbestartige Schuppung an Haaren und Kopfhaut
	*Atopisches Kopfhautekzem*
	Erytheme, Papulovesikel und Krusten („Milchschorf“) in der Regel ab dem 3. Lebensmonat

Die Grundlage jeder Psoriasisbehandlung bildet die topische Therapie mit begleitender Basistherapie, welche in den meisten Fällen zum Erfolg führen. Eine zumindest 1‑mal tägliche Anwendung wirkstofffreier Externa wird zum Schutz der Hautbarriere empfohlen. Je nach Ausmaß der Schuppung kann eine initiale Keratolyse angezeigt sein, um eine bessere Penetration der nachfolgend applizierten topischen Wirkstoffe gewährleisten zu können.

Topische Glukokortikoide der Klasse II und III werden als Wirkstoffe der ersten Wahl in der Initial- und Erhaltungstherapie der Psoriasis eingesetzt. Im Gesichts- und Genitalbereich sowie intertriginös sollten jedoch weniger potente Glukokortikoide für maximal 2 Wochen appliziert werden und im Anschluss durch topische Calcineurininhibitoren ersetzt werden [4]. Eine zunächst tägliche Applikation topischer Glukokortikoide mit langsamer Dosisreduktion und nachfolgender proaktiver Anwendung hat sich als zielführend erwiesen [1]. Kombinationsbehandlungen (Glukokortikoide plus Vitamin-D-Derivate) kommen als Erstlinientherapie zum Einsatz [1, 10].

Bei schweren Verläufen ist eine Systemtherapie aufgrund unzureichender Wirksamkeit lokaltherapeutischer Maßnahmen notwendig – hier kommen diverse Immunsuppressiva, Biologika und orale Retinoide zum Einsatz.

Als langjährig eingesetztes Medikament bei Erwachsenen als auch pädiatrischen PatientInnen wird Methotrexat (MTX) als Erstlinientherapie der mittelschweren und schweren Plaquepsoriasis empfohlen [1]. Zusätzlich weist MTX ein günstiges Wirkungsprofil bei der Psoriasisarthritis, schweren Verläufen einer Psoriasis guttata sowie pustulösen und erythrodermatischen Verläufen auf [8]. Bei ausschließlich pustulösen Psoriasisformen wird eine Behandlung mit Acitretin, einem aromatischen Retinoid der zweiten Generation, empfohlen [8].

Zur Therapie der Psoriasis im Kindes- und Adoleszentenalter sind die Biologika Adalimumab (ab dem 4. Lebensjahr) sowie Etanercept, Ustekinumab, Ixekizumab und Secukinumab (jeweils ab dem 6. Lebensjahr) zugelassenen [6, 9]. Für weitere Biologika werden derzeit Phase-III-Studien zur Therapie der pädiatrischen Psoriasis durchgeführt [9].

**Figure 3:**
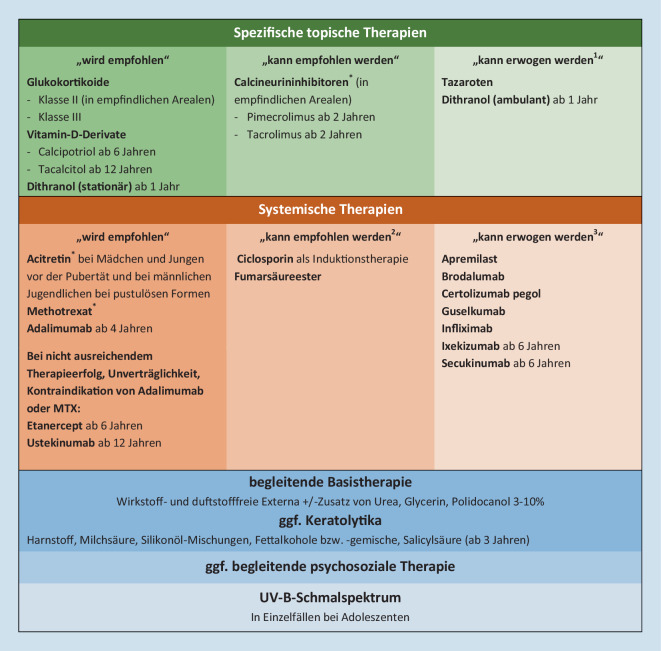

Regelmäßiges Screening auf mögliche kardiovaskuläre, metabolische als auch psychiatrische Komorbiditäten sowie eine frühzeitige Intervention und begleitende psychosoziale Unterstützung runden den Therapieplan ab [6]. Eine Übersicht der Therapieoptionen im Kindes- und Jugendalter ist in Abb. 3 angeführt.

## Fazit für die Praxis

- Die Plaquepsoriasis bildet die häufigste Erscheinungsform der Psoriasis vulgaris im Kindes- und Jugendalter.
- Die Diagnose wird in der Regel klinisch gestellt. Seltene Psoriasisvarianten und das Auftreten psoriatischer Hautveränderungen an atypischen anatomischen Lokalisationen erfordern eine Probebiopsie.
- In den meisten Fällen sind lokaltherapeutische Maßnahmen für einen Therapieerfolg ausreichend.
